# Protecting the healthcare workers in low- and lower-middle-income countries through vaccination: barriers, leverages, and next steps

**DOI:** 10.1080/16549716.2023.2239031

**Published:** 2023-07-27

**Authors:** Karla Kroflin, Mariana Gonzalez Utrilla, Michael Moore, Marta Lomazzi

**Affiliations:** a School of Medicine, University of Zagreb, Zagreb, Croatia; b World Federation of Public Health Associations, Geneva, Switzerland; c School of Health and Related Research, University of Sheffield, Sheffield, UK; d Institute of Global Health, University of Geneva, Geneva, Switzerland

**Keywords:** Vaccines, policy, health workers, health, prevention

## Abstract

Healthcare workers play a critical role in providing medical care to individuals and communities. Due to the nature of their work, compared to the general public, healthcare workers are at a higher risk of exposure to infectious diseases, including vaccine-preventable ones. The routine vaccination of healthcare workers in low- and lower-middle-income countries is a critical issue. Vaccination not only protects healthcare workers from contracting infectious diseases but also prevents the spread of diseases to the patients, reduces healthcare costs, increases healthcare workers’ morale and productivity, and demonstrates a commitment to health and safety. However, the implementation of policies for routine vaccination of healthcare workers in low- and lower-middle-income countries faces several challenges, including lack of funds, lack of evidence-based data, vaccination hesitancy through misguided beliefs, and low literacy among healthcare workers. In this article, we discuss the need for a policy for routine vaccination of healthcare workers in low- and lower-middle-income countries. We also analyse the barriers and recommendations for policy implementation and the role of partnerships. Additionally, we highlight the main points of the World Federation of Public Health Associations’ policy statement ‘Protecting the Healthcare Workforce in Low- and Middle-Income Countries through Vaccination’ which has the potential to drive policy-makers and healthcare organisations worldwide into prioritising routine vaccination of healthcare workers in low- and lower-middle-income countries.

## Why healthcare workers’ vaccination matters?

Healthcare workers (HCWs) play a vital role in providing medical care to individuals and communities. Due to the nature of their work, compared to the general public, they are at a higher risk of exposure to infectious diseases, including vaccine-preventable ones [1]. The World Health Organization (WHO) suggests the following vaccinations for HCWs: Bacillus Calmette–Guérin, diphtheria, hepatitis B, influenza, measles, meningococcal, pertussis, polio, rubella, severe acute respiratory syndrome coronavirus 2, and varicella [2]; yet, despite such clear guidelines, these are not always implemented, especially in less wealthy countries. Several well-recognised organisations are actively engaged in promoting the safety of HCWs across various domains. Among those, the World Federation of Public Health Associations (WFPHA), an international non-governmental organisation in an official relationship with the WHO, has for several years been advocating for the rights and concerns of millions of health professionals worldwide to protect them and the communities they serve [3].

Routine vaccination of HCWs not only protects them from contracting infectious diseases but also helps prevent the spread of diseases to their patients [4]. The prevention of outbreaks and control of the spread of infectious diseases are linked with patient safety. Routine vaccination of HCWs, therefore, can help reduce healthcare costs associated with treating infected individuals. By routinely vaccinating HCWs, healthcare organisations can reduce the risk of outbreaks that overburden health systems and increase costs [5,6]. Moreover, HCWs who are protected against vaccine-preventable diseases are less likely to miss work due to illness or infection which can improve confidence and increase overall productivity. This, in turn, is essential for providing quality healthcare and improving the efficiency of the overall healthcare system [6]. Similarly, vaccinated HCWs would be more confident in their ability to perform their duties as they are less likely to experience stress and anxiety related to their work [7]. With these possibilities in mind, WFPHA’s International Immunization Policy Taskforce undertook a series of focus group meetings with national public health associations and vaccination experts from low- and lower-middle-income countries (LICs and LMICs). The experts helped to gather different viewpoints that then determined the needs of health workers in these settings. They discussed the urgent need for policies specifically targeting health workers in LICs and LMICs. Thematic content analysis was conducted after focus group meetings in September 2022 and December 2022. The main barriers and recommendations that were discussed formed the basis of the official WFPHA policy ‘Protecting the Healthcare Workforce in Low- and Middle-Income Countries through Vaccination’ (Table 1).

**Table 1.** Main barriers and recommendations as highlighted in the WFPHA policy.

Main barriers from the WFPHA policy	Main recommendations from the WFPHA policy
Lack of funds	● Expand COVAX initiative to cover HCWs ● Partnerships with WHO, GAVI, governments, universities, and communities
Lack of vaccine availability	● Mandatory insurance for HCWs including vaccination ● Utilise COVID vaccination infrastructures to vaccinate HCW
Lack of evidence-based data	● Collect national data focusing on HCWs vaccination
Hesitancy, beliefs, and low literacy	● Increase awareness and literacy from the university partnering with health-related students organisations ● Advocacy at different levels: work locally promoting a change within hospitals, and nationally with government through effective lobbying
Lack of specific schedule for HCWs	● Specific schedules and funds addressed to HCWs (as done for children and pregnant women), HCWs vaccination as part of the Occupational Safety Plan

## Barriers from the World Federation of Public Health Associations policy

It is important to emphasise that the primary requirement for implementing the recommendations outlined below is the genuine commitment of governments to safeguard HCWs. Regrettably, numerous governments are yet to prioritise the protection of HCWs as essential for the well-being and prosperity of their nations. Consequently, this lack of prioritisation hinders the successful implementation of the proposed recommendations despite the potential for their positive outcomes.

The WFPHA policy aims to support national public health associations and professionals worldwide in their advocacy efforts with governments and other organisations. Routine vaccination of HCWs is in line with international standards set by organisations such as the WHO and the Centers for Disease Control and Prevention [2,8]. A policy for routine vaccination would send a clear message that the governments and healthcare organisations are committed to the health and safety of their workers and the community as a whole [9]. By implementing a policy for routine vaccination of HCWs, governments and organisations can encourage health workers to get vaccinated, thereby increasing overall vaccine uptake, helping to build herd immunity, and reducing the risk of outbreaks [10]. The WFPHA policy highlights the main barriers to be overcome such as the lack of funds, lack of specific schedules for HCWs, lack of evidence-based data, along with hesitancy, beliefs, and low literacy. All immunisations for health workers should ideally be provided free of charge to the recipient [2]. First, a shortage of funds is frequently a major obstacle in introducing good vaccine standards. For instance, in LICs and LMICs, it is common for HCWs to pay for their own vaccinations. Although The Global Alliance for Vaccines and Immunization (GAVI) has been a significant contributor, it is unable to provide all vaccinations for HCWs throughout all countries. In addition, the situation is often exacerbated by the lack of availability of appropriate vaccines. Second, there is a lack of research focusing specifically on vaccination among HCWs in LICs and LMICs. Policy-makers would greatly benefit from evidence-based research with a focus on disability-adjusted life years and quality-adjusted life years. Meta-analyses could also support implementation of necessary strategies [11]. Third, vaccination hesitancy of HCWs through religious beliefs and a lack of awareness and sensitisation are other key barriers that need to be overcome to ensure appropriate vaccine uptake. Lack of awareness and sensitisation were mentioned as barriers, since medical training in many countries does not provide enough education and opportunities in terms of vaccination. Even among trained health professionals, religious views in some countries have a significant impact on attitudes regarding vaccination; it is important to understand this factor and how it affects HCWs so that appropriate steps may be taken [12].

## Recommendations from the World Federation of Public Health Associations policy

Working in partnership is of utmost importance to overcome all those difficulties. The WHO is monitoring vaccination worldwide, but well-established partnerships are crucial for success. Organisations such as GAVI, World Bank, and United Nations Children’s Fund should be influenced by advocacy. To achieve the best results, the ministries that will manage the implementation of the policy should be involved from the beginning. Influential partnerships may facilitate national governments to adopt all recommendations. Where there have been reports of underperformance by the Ministries of Health, the WFPHA policy could help professionals to work in consultation with the local Ministry of Health to improve the situation. Additionally, policies might be promoted in national governments from two sides as part of an advocacy strategy. One side is working with the WHO, and the other one would be working with the WFPHA member associations. Moreover, the involvement of universities and schools of public health would improve advocacy efforts.

Addressing this issue should focus on guidelines where official policies and recommendations are especially applied to the immunisation of HCWs. Controlling diseases that can be prevented by vaccination requires a systematic strategy, such as having step-by-step guidelines. The first step could be for public health specialists to work with hospital directors to promote progress locally. Once governments have developed plans and policies, public health experts must be trained to lead implementation. Every government should make the immunisation of health professionals a top priority in its whole vaccination programme, even though it may be necessary to undertake certain policy changes to encourage HCWs to be vaccinated. Health professionals’ immunisation guidelines must differ from the national policy that impacts the entire population, as it is essential to create a vaccination programme specifically for healthcare professionals. The choice to prioritise routine immunisation of health workers, as with children and pregnant women, is one that requires political will. Health ministries within governments should make this choice. For this reason, pressure must be directed to national authorities through carefully designed lobbying activities.

Through its dedicated policy, WFPHA calls for guarantees for health professionals to be able to access free vaccinations similarly to children and pregnant women. It is essential for every government to make an ethical commitment to prioritise the protection of those individuals who protect the health of the jurisdiction, regardless of the financial investment involved.

The Expanded Immunization Programme of the WHO is a set of recommendations that names the target populations and vaccines that should be distributed [13]. A similar approach should be extended to health workers. Other alternatives include the vaccination of health workers in the occupational safety plan for the category of work or using the coronavirus disease 2019 (COVID-19) vaccination facilities already in place and reassigning them for this new purpose. Policy decisions are most strongly influenced by the dynamics of the health system, which in many nations have an impact on both public and private health sectors. The policy is determined by the partnerships available, how these are incorporated within each of these groups, and the type and availability of funding. One proposed funding strategy is to use the vaccines pillar of the Access to COVID-19 Tools Accelerator (COVAX) initiative in collaboration with GAVI to expand on programmes for vaccinating health workers against COVID-19 and other diseases. Making vaccine insurance available to all healthcare professionals and students is another strategy that could be implemented. However, efforts on education should be managed in parallel commencing as soon as possible at the university level with mentorship initiatives used to strengthen literacy and awareness levels. Collaborating with the International Federation of Medical Students Associations, for example, and organisations representing students of other health disciplines might be effective. It appears that stakeholders are aware of the necessity of immunising HCWs; yet, this issue is frequently not given high priority. Vaccination programmes, policies, and standards could be developed, modified, and put into place based on the WFPHA policy. Advocacy by the WFPHA and its member public health associations working with academics and other health professionals is unquestionably needed.

## Paper context

HCWs are at a higher risk of exposure to infectious diseases. The vaccination of HCWs in LICs and LMICs is a critical issue, and the implementation of policies for routine vaccination faces many challenges. We discuss the WFPHA policy statement ‘Protecting the Healthcare Workforce in Low- and Middle-Income Countries through Vaccination’ which has the potential to drive policy-makers and healthcare organisations in prioritising routine vaccination of HCWs worldwide.

## References

[1] Sepkowitz KA, Eisenberg L. Occupational deaths among healthcare workers. Emerg Infect Dis. 2005;11:1003–4. doi: 10.3201/eid1107.041038 16022771 PMC3371777

[2] World Health Organization. Implementation guide for vaccination of health workers. Geneva: World Health Organization; 2022.

[3] World Federation of Public Health Associations. [cited 2023 Jul 13]. Available from: https://www.wfpha.org/

[4] Billings J, Ching BCF, Gkofa V, Greene T, Bloomfield M. Experiences of frontline healthcare workers and their views about support during COVID-19 and previous pandemics: a systematic review and qualitative meta-synthesis. BMC Health Serv Res. 2021 Sep 6;21:923. 34488733 10.1186/s12913-021-06917-z PMC8419805

[5] Pople D, Monk EJM, Evans S, Foulkes S, Islam J, Wellington E, et al. Burden of SARS-CoV-2 infection in healthcare workers during second wave in England and impact of vaccines: prospective multicentre cohort study (SIREN) and mathematical model. BMJ. 2022 Jul 20;378:e070379. doi: 10.1136/bmj-2022-070379 35858689 PMC9295077

[6] Largeron N, Lévy P, Wasem J, Bresse X. Role of vaccination in the sustainability of healthcare systems. J Mark Access Health Policy. 2015 Aug 12;3:27043. doi: 10.3402/jmahp.v3.27043 PMC4802702 27123188

[7] Thorsteinsdottir B, Madsen BE. Prioritizing health care workers and first responders for access to the COVID-19 vaccine is not unethical, but both fair and effective – an ethical analysis. Scand J Trauma Resusc Emerg Med. 2021 Jun 4;29:77. doi: 10.1186/s13049-021-00886-2 34088336 PMC8177265

[8] Centers for Disease Control and Prevention. Recommended Vaccines for Healthcare Workers. [cited 2023 Mar 12]. Available from: https://www.cdc.gov/vaccines/adults/rec-vac/hcw.html

[9] Kruk ME, Gage AD, Arsenault C, Jordan K, Leslie HH, Roder-DeWan S, et al. High-quality health systems in the sustainable development goals era: time for a revolution. Lancet Glob Health. 2018 Nov;6:e1196–e1252. 30196093 10.1016/S2214-109X(18)30386-3 PMC7734391

[10] Anderson RM, May RM. Vaccination and herd immunity to infectious diseases. Nature. 1985;318:323–329. doi: 10.1038/318323a0 3906406

[11] Mardani RAD, Wu WR, Nhi VT, Huang HC. Association of breastfeeding with undernutrition among children under 5 years of age in developing countries: a systematic review and meta-analysis. J Nurs Scholarsh. 2022 Nov;54:692–703. doi: 10.1111/jnu.12799 35844158

[12] Njoga EO, Mshelbwala PP, Abah KO, Awoyomi OJ, Wangdi K, Pewan SB, et al. COVID-19 vaccine hesitancy and determinants of acceptance among healthcare workers, academics and tertiary students in Nigeria. Vaccines. 2022;10:626. doi: 10.3390/vaccines10040626 35455375 PMC9032510

[13] World Health Organization: Essential Programme on Immunization. [cited 2023 Jul 13]. Available from: https://www.who.int/teams/immunization-vaccines-and-biologicals/essential-programme-on-immunization

